# Conformational dynamics during high-fidelity DNA replication and translocation defined using a DNA polymerase with a fluorescent artificial amino acid

**DOI:** 10.1074/jbc.RA120.016617

**Published:** 2020-12-10

**Authors:** Tyler L. Dangerfield, Kenneth A. Johnson

**Affiliations:** Institute for Cellular and Molecular Biology, Department of Molecular Biosciences, University of Texas, Austin, Texas, USA

**Keywords:** bacteriophage T7, DNA polymerase, DNA replication, conformational change, fluorescent unnatural amino acid, translocation, pyrophosphorolysis, pre-steady-state kinetics, global data fitting, free energy profile., BSA, bovine serum albumin, PEI, polyethyleneimine

## Abstract

We address the role of enzyme conformational dynamics in specificity for a high-fidelity DNA polymerase responsible for genome replication. We present the complete characterization of the conformational dynamics during the correct nucleotide incorporation forward and reverse reactions using stopped-flow and rapid-quench methods with a T7 DNA polymerase variant containing a fluorescent unnatural amino acid, (7-hydroxy-4-coumarin-yl) ethylglycine, which provides a signal for enzyme conformational changes. We show that the forward conformational change (>6000 s^−1^) is much faster than chemistry (300 s^−1^) while the enzyme opening to allow release of bound nucleotide (1.7 s^−1^) is much slower than chemistry. These parameters show that the conformational change selects a correct nucleotide for incorporation through an induced-fit mechanism. We also measured conformational changes occurring after chemistry and during pyrophosphorolysis, providing new insights into processive polymerization. Pyrophosphorolysis occurs via a conformational selection mechanism as the pyrophosphate binds to a rare pretranslocation state of the enzyme–DNA complex. Global data fitting was achieved by including experiments in the forward and reverse directions to correlate conformational changes with chemical reaction steps. This analysis provided well-constrained values for nine rate constants to establish a complete free-energy profile including the rates of DNA translocation during processive synthesis. Translocation does not follow Brownian ratchet or power stroke models invoking nucleotide binding as the driving force. Rather, translocation is rapid and thermodynamically favorable after enzyme opening and pyrophosphate release, and it appears to limit the rate of processive synthesis at 4 °C.

DNA polymerases are ideal systems to study enzyme specificity because fidelity is physiologically important and alternative substrates (noncognate base pairs) are well known. DNA polymerases alter their substrate specificity during each cycle of template-dependent nucleotide incorporation, allowing efficient selection of the correct nucleotide over the structurally similar mismatched nucleotides (1). Selection of the correct nucleotide is assisted by base pairing of the incoming nucleotide with the templating base. However, the small free energy differences between base pairing for correct *versus* incorrect bases cannot account for the extremely high specificity of these enzymes (2), although hydrogen bonds do enforce base-pair geometry that contributes to satisfying steric requirements (3). Proofreading mechanisms further improve overall accuracy, but exonuclease specificity is dictated by the binding of the primer/template at the polymerase site to modulate transfer to the exonuclease site (4). DNA polymerases vary widely from low-fidelity repair enzymes (5, 6, 7, 8, 9) to high-fidelity enzymes necessary for genome replication (10, 11, 12). It is surprising that high-fidelity enzymes catalyze replication at faster rates than low-fidelity enzymes, raising additional questions regarding the molecular mechanisms responsible for enzyme specificity.

DNA polymerase from bacteriophage T7 provides a simple enzyme capable of fast and accurate DNA replication. Crystal structures of the T7 DNA polymerase (Fig. 1) show large structural rearrangements, particularly in the “fingers” domain, upon nucleotide binding (13). In a minimal model (Equation 1), the enzyme–DNA complex with a primer strand n-nucleotides in length (*ED*_*n*_) binds nucleotide (*N*) in the open state. A transition to the closed state (*ED*_*n*_*N → FD*_*n*_*N*) then facilitates the chemical reaction followed by enzyme opening and release of pyrophosphate (*PP*_i_).

(1) $$ED_{n}+N\overset{K_{1}}{⇄}ED_{n}N\overset{k_{2}}{\underset{k_{-2}}{⇄}}FD_{n}N\overset{k_{3}}{\underset{k_{-3}}{⇄}}FD_{n+1}.PP_{i}\overset{k_{4}}{\underset{k_{-4}}{⇄}}ED_{n+1}+PP_{i}$$

**Figure 1:**
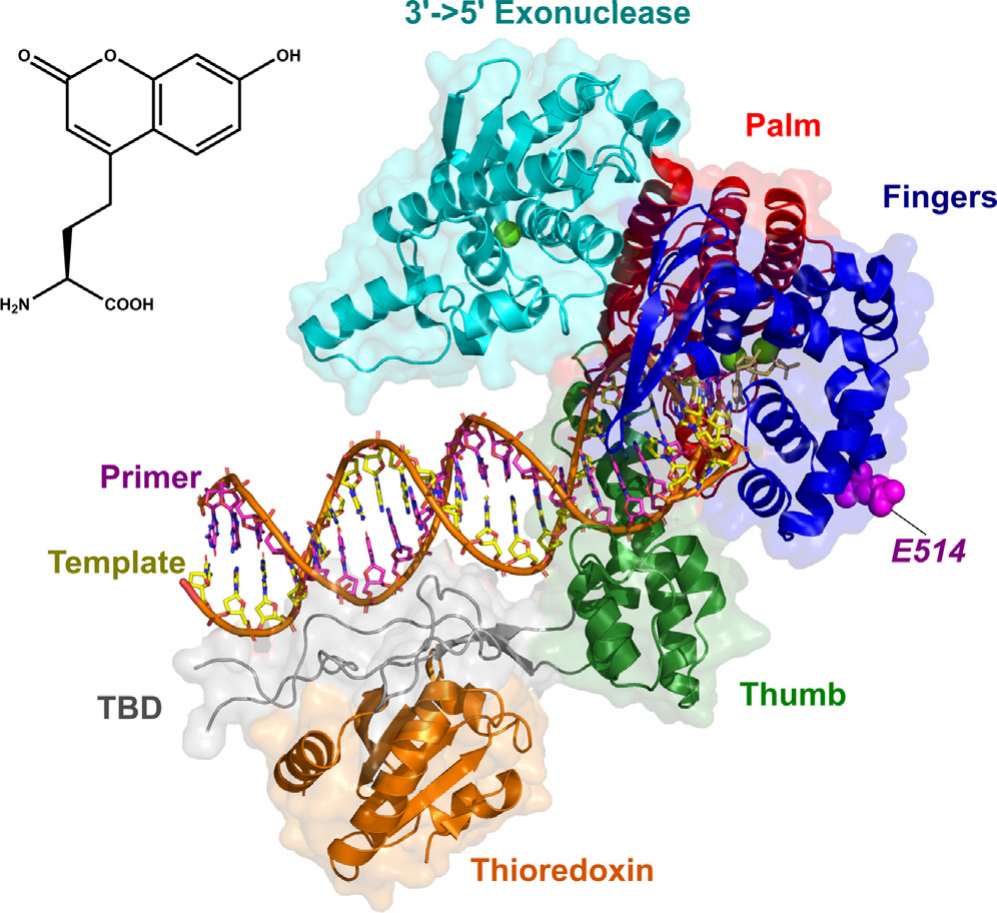
**Structure of fluorescent unnatural amino acid and T7 DNA polymerase.***Top left*, structure of the fluorescent unnatural amino acid, L-(7-hydroxycoumarin-4-yl) ethylglycine (7-HCou). *Right*, structure of T7 DNA polymerase: The overall structure resembles a right hand with the polymerase active site situated at the interface of the palm domain (*red*), fingers domain (*blue*), and thumb domain (*green*). 7-HCou is inserted at position 514, on the back side of the fingers domain and is shown as *magenta spheres*. Thioredoxin (*orange*) is a 12-kDa host redox protein that associates with T7 gene product 5 through a unique extension of the thumb domain called the thioredoxin binding domain (TBD, *gray*) and helps with stable primer (*magenta*)/template (*yellow*) DNA binding. The 3’-5’ proofreading exonuclease domain (*cyan*) increases the fidelity of the enzyme by at least a factor of 1000. Magnesium ions are shown as *green spheres* in the exonuclease and polymerase active sites. The structure was drawn using Pymol from PDBID: 6p7e (41).

Similar conformational changes have been observed for both high- and low-fidelity polymerases, but the contribution of these prechemistry steps to enzyme specificity has been controversial, leaving many questions unanswered. What role does this conformational change play in selecting the correct nucleotide over the three incorrect but structurally similar substrates? Is the rate of the conformational change faster or slower than the chemistry and how does this affect the fidelity? How do the kinetics of the conformational change differ when comparing low- and high-fidelity DNA polymerases?

Initial attempts to look for a rate-limiting conformational change were based on examining the effect of sulfur substitution of one of the nonbridging oxygens of the incoming nucleotide, ostensibly to slow the rate of chemical reaction. The absence of a thio-elemental effect on the measured rate of incorporation was used to argue for a rate-limiting conformational change preceding chemistry (11, 14). However, subsequent studies showed a much smaller-than-anticipated inherent elemental effect (15) and studies on DNA polymerase β provided evidence for a fast conformational change preceding chemistry (16). Accordingly, the rate of the conformational change was deemed to be unimportant for fidelity other than contributing to the fraction of enzyme in the closed state to facilitate catalysis. However, subsequent measurements using higher-fidelity enzymes led to a new paradigm for understanding the role of fast conformational changes in fidelity based on kinetic partitioning of the closed *FD*_*n*_*.N* state (1). Essentially, the conformational change locks a correct nucleotide at the active site to commit it to the forward reaction, but the enzyme rapidly opens to afford release rather than incorporation of a mismatch. Further studies on HIV reverse transcriptase provided more complete data to support this model (17), but it still remained controversial (18) in spite of support from molecular dynamics simulation methods (19, 20).

There is little question that the change in structure from an open to a closed state leads to alignment of catalytic residues, and that certainly influences the rate of the chemical reaction. However, when the conformational change is faster than the chemistry, the question as to whether the structural change directly influences specificity must be based on complete kinetic analysis of the pathway. Because of the disparate results obtained for low- and moderate-fidelity polymerases, it is crucial that the role of the conformational change be defined for a high-fidelity enzyme. Previous attempts to examine the kinetics of the conformational change using T7 DNA polymerase were flawed because they were based on constructing a cys-lite mutant to afford site-specific labeling with a fluorescent probe (1). Unfortunately, the cys-lite variant containing nine mutations reduced the fidelity and stability of the enzyme. Here we take advantage of new methods to site specifically label T7 DNA polymerase with a fluorescent unnatural amino acid, (7-hydroxy-4-coumarin-yl) ethylglycine, under conditions where the fluorescent variant retains the fidelity and reaction kinetics of the wildtype enzyme (21). Unless noted otherwise, all experiments were performed with the labeled enzyme.

Prior measurements of translocation using single molecule methods (22, 23) produced data that suggest a rapid equilibrium constant for translocation with an equilibrium constant near unity, so that dNTP binding will trap the DNA in the translocated state. However, it is important to note that single molecule measurements at equilibrium do not necessarily reflect the population of intermediates formed during processive synthesis. Here we provide a unique solution to the problem by determining the rate constants for translocation during synthesis as part of global data fitting.

We use the coumarin fluorescence signal to measure the rates of the forward and reverse conformational change steps for the enzyme in concert with measurements of the rates of polymerization. Corresponding measurements for the reverse reaction (pyrophosphorolysis) and global data fitting of all experiments provides well-constrained rate constants to define a complete free-energy profile for the reaction pathway, including DNA translocation.

## Results

### Kinetics and equilibrium of nucleotide binding

We first measured the kinetics and equilibrium for nucleotide binding steps (*K*_*1*_, *k*_*2*_, and *k*_−*2*_ in Equation 2) using a 2’,3’ dideoxy-nucleotide-terminated primer to allow dATP binding but prevent the chemical reaction.

(2) $$ED_{dd}+dATP\overset{K_{1}}{⇄}ED_{dd}dATP\overset{k_{2}}{\underset{k_{-2}}{⇄}}FD_{dd}dATP$$

A 27-nt (nucleotide) 2’,3’-dideoxy-terminated primer strand was annealed to a 45-nt template strand (27_dd_/45, denoted as *D*_*dd*_) and then incubated with the labeled enzyme to form the *ED*_*dd*_ complex. Initial experiments performed at 20 °C indicated that the rate of the conformational change (*k*_*2*_) was too fast to measure in the stopped flow except at very low concentrations of nucleotide. To better estimate the rate of the conformational change, as well as other steps in the pathway that are too fast to measure accurately at 20 °C, we opted to perform all experiments in this paper at 4 °C.

#### dATP binding kinetics

**Figure 2:**
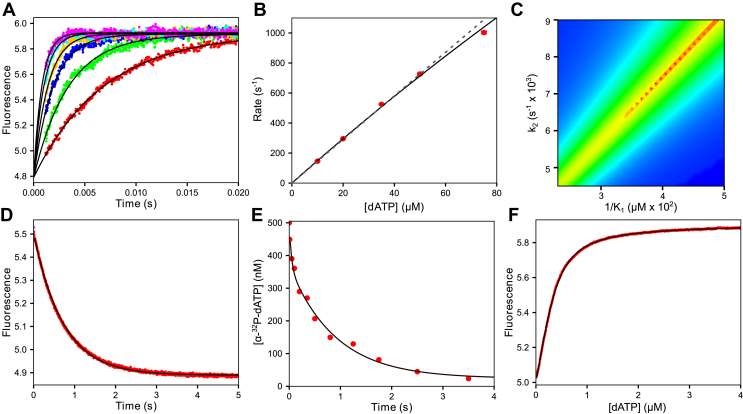
**Kinetics and equilibrium of nucleotide binding steps preceding chemistry.***A*, stopped flow dATP binding rate. T7 DNA polymerase E514Cou (750 nM), thioredoxin (15 μM), and DNA_dd_ (1 μM) were mixed with dATP (5–100 μM) and Mg^2+^ (12.5 mM) to start the reaction. *Colored dots* are stopped flow traces at various dATP concentrations, and *black lines* are single exponential fits to the data. *B*, decay rate *versus* [dATP]. Rates are derived from the single exponential fits to the data in (*A*). The *dashed gray line* through the data points is the fit to a linear function with a y-intercept of 0 and a slope of 14.5 ± 0.3 μM^−1^ s^−1^. The *solid black line* through the data is the fit to a hyperbola (*k*_max_: 12,200 ± 6850 s^−1^, *K*_*d,app*_: 805 ± 470 μM). Only data up to 75 μM are shown because the reaction was complete in the dead time of the stopped flow instrument at higher concentrations. *C*, 2D FitSpace calculation. The relationship between ground-state binding (1/*K*_*1*_) and the conformational change step (*k*_*2*_) is shown in terms of χ^2^ from the 2D FitSpace calculation in KinTek Explorer. The *red area* of the graph shows well-constrained rate constant combinations, whereas areas in *green* and *blue* are not well constrained. This experiment sets a lower limit of 6000 s^−1^ for the rate of the conformational change at a 0.99 χ^2^ confidence threshold and shows the linear relationship between *k*_*2*_ and 1/*K*_*1*_. *D*, fluorescence measurement of dATP dissociation rate. T7 DNA polymerase E514Cou (500 nM), thioredoxin (10 μM), DNA_dd_ (600 nM), and dATP (500 nM) were mixed with wildtype T7 DNA polymerase (10 μM), thioredoxin (50 μM), and 27/45 DNA (with a 3’-OH group, 10 μM), which serves as a trap for nucleotide released from the ternary T7 DNA polymerase E514Cou-DNA_dd_-dATP complex. The *black line* through the data is the best fit to a single exponential function, with an observed rate of 1.39 ± 0.002 s^−1^. *E*, quench flow measurement of dATP dissociation rate. T7 DNA polymerase E514Cou (500 nM), thioredoxin (5 μM), DNA_dd_ (500 nM), BSA (0.1 mg/ml), and [α-^32^P]-dATP (500 nM) were mixed with wildtype T7 DNA polymerase (5 μM), thioredoxin (20 μM), and 27/45 DNA (5 μM) to serve as a free nucleotide trap. The *black line* through the data is the best fit to a double exponential function, where the small initial fast phase corresponds to unbound [α-^32^P]-dATP incorporated rapidly into the trap DNA and the second slower phase corresponding to the nucleotide dissociation rate has a rate of 1.25 ± 0.15 s^−1^. *F*, equilibrium dATP binding titration. A solution of T7 DNA polymerase E514Cou (400 nM), thioredoxin (8 μM), DNA_dd_ (600 nM), and Mg^2+^ (12.5 mM) was titrated with dATP from a Hamilton syringe while monitoring fluorescence over the course of the 5-min titration with constant stirring. The signal was corrected for the small dilution and fit to a quadratic equation (*black line*) giving *K*_*d,dATP*_: 107 ± 1 nM and an enzyme concentration of 400 ± 1 nM.

The nucleotide binding rate was measured by mixing a preformed labeled E-DNA_dd_ complex with various concentrations of dATP (5–100 μM) and Mg^2+^ in the stopped flow (Fig. 2*A*). Although a good signal could be obtained by direct excitation of the coumarin label, a larger signal with the same kinetics was obtained when fluorescence emission from the coumarin was excited by FRET from tryptophan residues in the enzyme. An increase in fluorescence was observed and fit well to a single exponential function (Equation 3) at concentrations giving a measurable signal (up to 100 μM). The exponential function was:

(3) $$y=A_{0}+A_{1}\cdot \left(1-e^{-{\text{λ}}_{1}t}\right)$$

where *A*_*0*_ is the starting fluorescence, *A*_*1*_ is the amplitude, λ_*1*_ is the observed decay rate, and *t* is time.

The plot of the observed rate at each dATP concentration (Fig. 2*B*) can be fit using a straight line (dashed gray line in Fig. 2*B*) or a hyperbola (Equation 4, solid black line in Fig. 2*B*). The model in Equation 2 predicts a hyperbolic concentration dependence of the observed rate.

(4) $$rate=\frac{k_{\max}\left[dATP\right]}{K_{d}+\left[dATP\right]}=\frac{K_{1}k_{2}\left[dATP\right]}{1+K_{1}\left[dATP\right]}+k_{-2}$$

The slope of the linear fit was 14.5 ± 0.3 μM^−1^ s^−1^, which defines the second-order rate constant for nucleotide binding (*K*_*1*_*k*_*2*_) leading to the *FD*_*dd*_*N* state (Equation 2). A slightly better fit to the data was obtained assuming a rapid-equilibrium ground-state dATP binding (*K*_*1*_) followed by a conformational change step (*k*_*2*_). Conventional analysis of this data set using the two-step binding model reveals the large errors on the extrapolated maximum rate (*k*_*2*_ +*k*_−*2*_ = 12,200 ± 6850 s^−1^) and the *K*_*d*_ *= 1/K*_*1*_ (805 ± 470 μM). The initial slope of the concentration dependence provides an accurate estimate for *K*_*1*_*k*_*2*_ for the two-step model, even though neither *K*_*1*_ nor *k*_*2*_ is well defined.

Fitting the primary experimental data globally using KinTek Explorer including confidence contour analysis (24) provided a well-defined lower limit on *k*_*2*_ ≥ 6000 s^−1^ as well as a well-defined value for *K*_*1*_*k*_*2*_ (Fig. 2*C*) shown by the linear relationship between *k*_*2*_ and 1/*K*_*1*_ in the red line of the 2D confidence contour plot. With the equation-based data fitting shown here, the raw data were fit using a total of 17 parameters and the trace at the highest concentration was omitted because of the low amplitude and large errors. In contrast, with global fitting the full set of primary data was fit using only two parameters. Global data fitting provides a more realistic assessment of errors in fitting and gives lower limits for parameters that are not well constrained.

This is the first of many examples of the advantages of using global data fitting to reveal the information content of kinetic data. As experiments get more complex and interrelated, we emphasize the importance of interpreting experimental data globally using a single unifying model to overcome the traditional piecemeal approach of fitting using simplified equations for complex mechanistic studies. Although we favor the fit by simulation, here we initially present plots of rate *versus* concentration followed by quantitative analysis of the concentration dependence of the observed rate (when possible) to reveal the underlying mechanism. The equation-based data fitting provides a rationale to explain our interpretation of the results and provides the basis for developing a model that is then used to fit data globally.

#### dATP dissociation rate experiments

To more accurately measure the rate constant for nucleotide dissociation (limited by enzyme opening, *k*_−*2*_), a stopped flow experiment was performed where a solution containing a ternary *E.DNA*_*dd*_*.dATP* complex was mixed with a large excess of unlabeled wildtype *E.DNA* complex to start the reaction. The large excess of wildtype *E.DNA* complex (with a normal 3’OH primer) was used to trap dATP released from the labeled *E.DNA*_*dd*_*.dATP* ternary complex. This mixture works efficiently as a trap for free dATP because the rate of nucleotide binding and incorporation by the large excess of wildtype enzyme is much faster than the slow release of the nucleotide from the labeled *E.DNA*_*dd*_*.dATP* ternary complex. The decrease in fluorescence (Fig. 2*D*) fits a single exponential function with an observed decay rate of 1.39 ± 0.002 s^−1^. Since the kinetics of nucleotide binding and incorporation are known for the wildtype enzyme (21), these parameters can be used when fitting by simulation to determine the nucleotide dissociation rate, even if the nucleotide trap is not 100% effective.

To ensure the dissociation rate measured in the stopped-flow experiment indeed reflected the nucleotide dissociation rate and not an artifact of the fluorescence signal, a similar experiment was performed using rapid-quench methods to measure the nucleotide dissociation rate (Fig. 2*E*).

An *E.DNA*_*dd*_*.[α-*^*32*^*P]dATP* ternary complex was mixed with a large excess of wildtype *E.DNA* complex to start the reaction. Dissociation of the [α-^32^P]-dATP from the *ED*_*dd*_*.[α-*^*32*^*P]dATP* complex was monitored by the decrease in [α-^32^P]-ATP concentration as it binds to the wildtype enzyme and is converted to DNA product. The reaction was quenched by mixing with EDTA from the quench syringe at various times, and samples were analyzed by TLC on polyethyleneimine (PEI)-cellulose plates to separate the free nucleotide from the nucleotide incorporated into DNA 27/45 by the wildtype enzyme in the trap mixture. The data fit a double exponential—modeling the experiment by simulation provided a rationale for understanding the biphasic data. The small initial fast phase is due to the small amount of unbound nucleotide from the *E.DNA*_*dd*_*.dATP* mixture that is rapidly incorporated by the wildtype polymerase upon mixing, while the slower phase reflects the nucleotide dissociation rate, which is limited by the reverse of the conformational change step (*k*_−*2*_) from the labeled enzyme. The slower phase has a rate of 1.25 ± 0.15 s^−1^, which is in agreement with the stopped flow dissociation rate measurement.

#### Equilibrium dATP titration

An equilibrium titration of the labeled *E.DNA*_*dd*_ complex was performed with an increasing dATP concentration to get an independent measurement of the *K*_*d*_ for dATP binding (Fig. 2*F*). A solution of labeled *E.DNA*_*dd*_ was incubated in a cuvette inside a temperature-controlled cuvette in the titration module for the stopped flow. The titrant dATP was added to the cuvette over the course of 5 min with constant stirring at 4 °C while continuously monitoring the fluorescence (Fig. 2*F*). After correcting the fluorescence intensity for the small dilution during the titration, the resulting curve was fit using a quadratic equation (below):

$$y=F_{0}+\text{Δ}F\cdot \frac{{\left[ED\right]}_{0}+[N]+K_{d}-\sqrt{\left({{\left[ED\right]}_{0}+[N]+K_{d})}^{2}-4\cdot [ED][N\right]}}{2\cdot {\left[ED\right]}_{0}}$$

where F_0_ is the initial fluorescence, ΔF is the change in fluorescence upon saturation of dATP binding to the enzyme, [*ED*]_0_ is the initial concentration of the labeled enzyme *E.DNA*_*dd*_ complex, and [*N*] is the concentration of dATP added during the titration. The titration defines the active enzyme concentration, 400 ± 0.2 nM and *K*_*d*_ = 107 ± 1 nM.

The net *K*_*d,net*_ determined from the rate constants for the fit to the on- and off-rate experiments for a two-step binding model is defined by Equation 5.

(5) $$K_{d,net}=\frac{1}{K_{1}\left(1+K_{2}\right)}≃\frac{1}{K_{1}K_{2}}=\frac{k_{-2}}{K_{1}k_{2}}=96\pm 2\;nM$$

Because *K*_*2*_ >> 1, the net *K*_*d,net*_ is simply the ratio of the measured dissociation rate (*k*_−*2*_) divided by the observed second-order rate constant for nucleotide binding (*K*_*1*_*k*_*2*_). The calculated value is close to the value determined from the direct equilibrium titration experiment, indicating that all kinetically significant steps have been included for nucleotide binding.

### Kinetics of polymerization

**Figure 3:**
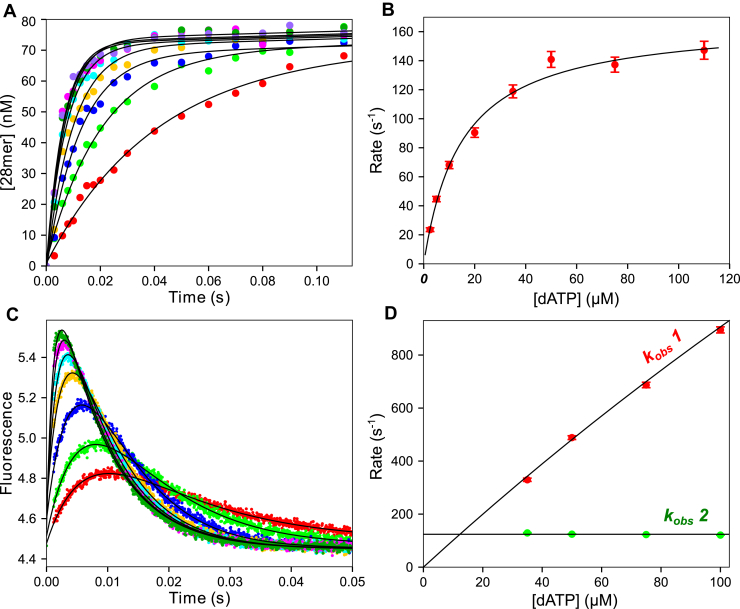
**Kinetics of dATP incorporation.***A*, quench flow pre-steady-state burst experiment. T7 DNA polymerase E514Cou (120 nM), BSA (0.1 mg/ml), thioredoxin (2.4 μM), and FAM-27/45 DNA (200 nM) were mixed with dATP (2.5–110 μM) and Mg^2+^ (12.5 mM) to start the reaction. Data at various dATP concentrations are shown as different colors, fit to a burst equation (*black line*) at each concentration. *B*, rate *versus* dATP concentration. Rates are from the fit of the exponential phase in (*A*) and are shown fit to a hyperbola (*black line*), estimating the maximum rate, *k*_*pol*_ = 210 ± 15 s^−1^ and *K*_*d,app*_ =15.5 ± 2 μM. *C*, stopped flow dATP incorporation. T7 DNA polymerase E514Cou (750 nM), thioredoxin (15 μM), and DNA 27/45 (1 μM) were mixed with dATP (5–100 μM) and Mg^2+^ (12.5 mM) to start the reaction. Data at each dATP concentration were fit to a double exponential function (*black lines*). *D*, rate *versus* dATP concentration for stopped flow fluorescence. Rates are from the data in (*C*). The fast phase data (*red points*, *k*_obs_ 1) were fit to a hyperbola to estimate the maximum rate of the conformational change (*k*_*2*_), 7800 ± 3400 s^−1^. The slow phase data (*green points*, *k*_obs_ 2) were fit to a line, setting a lower limit on the maximum rate of the isomerization following chemistry (*k*_*4*_) of 126 ± 4 s^−1^.

Next, experiments were performed using the DNA 27/45 substrate containing a 3’-OH on the primer strand to allow measurement of the rates of the covalent bond formation between the 3’-OH group of the primer strand and the α-phosphate of the incoming dNTP. A pre-steady-state burst experiment was performed using rapid-quench-flow methods (Fig. 3*A*). A preformed E.DNA complex was mixed with varying concentrations of dATP (2.5–110 μM) and Mg^2+^ to start the reaction. At various times, the reaction was quenched by mixing with EDTA, and the products were resolved by denaturing PAGE. For conventional data analysis, data at each dATP concentration were fit to a burst equation (Equation 6):

(6) $$y=A_{1}\cdot \left(1-e^{-{\text{λ}}_{1}t}\right)+k_{ss}t$$

where *A*_*1*_ is the amplitude of the exponential phase, λ_*1*_ is the rate of the exponential phase and *k*_*ss*_ is the slope of the linear phase. A hyperbolic fit of the concentration dependence of the rate of the exponential phase (Fig. 3*B*) provided estimates for the parameters from the maximum observed rate, *k*_*pol*_ = 210 ± 15 s^−1^, and half-maximal nucleotide concentration to define an *apparent K*_*d*_, *K*_*d,app*_ = 15.5 ± 2 μM. The ratio *k*_*pol*_*/K*_*d,app*_ = 13 ± 2 μM^−1^ s^−1^ provides an estimate of *k*_*cat*_*/K*_*m*_, the specificity constant for the first incorporation.

Note that the *K*_*d,app*_ measured in this experiment is ∼150 times larger than the value reported from the equilibrium titration experiment (*K*_*d*_ ∼100 nM), reflecting the fact that this experiment gives a value that is not the true *K*_*d*_ for dATP binding. This feature is attributable to the two-step binding kinetics that do not reach equilibrium on the timescale of a single turnover (17). The meaning of *k*_*pol*_ and *K*_*d,app*_ with respect to individual rate constants cannot be determined without more information, as described below in global data fitting including multiple experiments.

#### Time dependence of fluorescence changes during dATP incorporation

The next experiment was designed to monitor the changes in fluorescence in the stopped flow to measure the conformational changes involving enzyme closing after nucleotide binding and reopening after chemistry. A labeled *E.DNA* complex was mixed with various concentrations of dATP (5–100 μM) and Mg^2+^ to start the reaction. As shown in Figure 3*C*, we observed an increase in fluorescence during dATP binding followed by a slower decrease in fluorescence back to the starting value.

At all concentrations, the traces can be fit using a double exponential function (Equation 7):

(7) $$y=A_{0}+A_{1}\cdot \left(1-e^{-{\text{λ}}_{1}t}\right)+A_{2}\cdot \left(1-e^{-{\text{λ}}_{2}t}\right)$$

where *A*_*0*_ is the initial fluorescence, whereas *A*_*1*_ and *λ*_*1*_, and *A*_*2*_ and *λ*_*2*_ are the amplitudes and rates of the fast and slow phases, respectively.

The dATP concentration dependence of the rates of the fast and slow phases (Fig. 3*D*) shows an almost linear increase in the rate of the fast phase with increasing dATP concentrations and a constant observed rate for the slower phase at 126 ± 4 s^−1^. Fitting the concentration dependence of the fast phase to a hyperbola in an attempt to extract the maximum rate of the conformational change (*k*_*2*_) has large errors (7800 ± 3400 s^−1^) but is comparable with the values obtained for the nucleotide binding-rate experiment with DNA_dd_ in Figure 2*A*.

The increase in fluorescence is a function of the transition from open to closed enzyme (*ED*_*n*_*N* → *FD*_*n*_*N*), whereas the subsequent decrease is due to the closed to open transition (*FD*_*n+1*_*.PP*_*i*_ → *ED*_*n+1*_) after the chemical reaction (Equation 1). We know that the increase in fluorescence is correlated with enzyme structural changes seen after nucleotide binding to dideoxy-terminated DNA (Fig. 2). Moreover, global data fitting in correlating the fluorescence transients with the measured rates of the chemical reaction establish the pathway. Since *k*_*pol*_ measured in the quench flow burst experiment is approximately 200 s^−1^, the slower phase of the fluorescence transient (approximately 125 s^−1^) must be attributed to an isomerization step (*k*_*4*_) following chemistry that may be coincident with PP_i_ release (Equation 1).

Globally fitting these data along with nucleotide binding kinetics can afford estimates of *k*_*3*_, *k*_−*3*_, and *k*_*4*_ from the concentration dependence of the rate and amplitude of the pre-steady-state burst of incorporation (Fig. 3*A*) and the fluorescence transients (Fig. 3*C*). Simultaneous fitting of rates and amplitudes of the reactions allows resolution of *k*_*3*_, *k*_−*3*_, and *k*_*4*_, which we will show after all experiments have been included. Understanding global data fitting does not translate easily to equation-based data fitting where the rates and amplitudes are derived independently and using five independent parameters per trace lead to increased errors. Global data fitting affords direct estimates of the intrinsic rate constants while accounting for rates and amplitudes simultaneously. Moreover, the most accurate estimates are based on the inclusion of data from pyrophosphorolysis. Therefore, we postpone showing the global data fitting until all data have been presented.

The model invoking slow PP_i_ release following fast chemistry raises two testable hypotheses: 1) the slow PP_i_ release may limit the rate of polymerization during multiple nucleotide incorporations, which can be measured directly; 2) the reverse rate of chemistry (*k*_−*3*_) can be extracted from analysis of the pyrophosphorolysis reaction to define the internal equilibrium constant for chemistry, *K*_*3*_. These hypotheses will be tested by pyrophosphorolysis experiments in the following section.

### Kinetics of pyrophosphorolysis

Initial attempts to measure pyrophosphorolysis using 6-carboxyfluorescein labeled DNA (FAM-DNA) resolved on DNA sequencing gels (not shown) produced many bands, both above and below the starting 27-nt primer, making the experiment difficult to interpret. Owing to the high efficiency of polymerization, any dNTP produced during the slower pyrophosphorolysis reaction becomes a substrate for the primer extension reaction. To circumvent the difficulties with this experimental approach, we used a previously reported strategy (14) that required a DNA substrate containing [α-^32^P]-dNMP incorporated enzymatically at the 3’ end of the primer strand of DNA 27/45 (named [α-^32^P]-28/45) as described in the experimental procedures section. This afforded direct measurement of a single round of pyrophosphorolysis.

**Figure 4:**
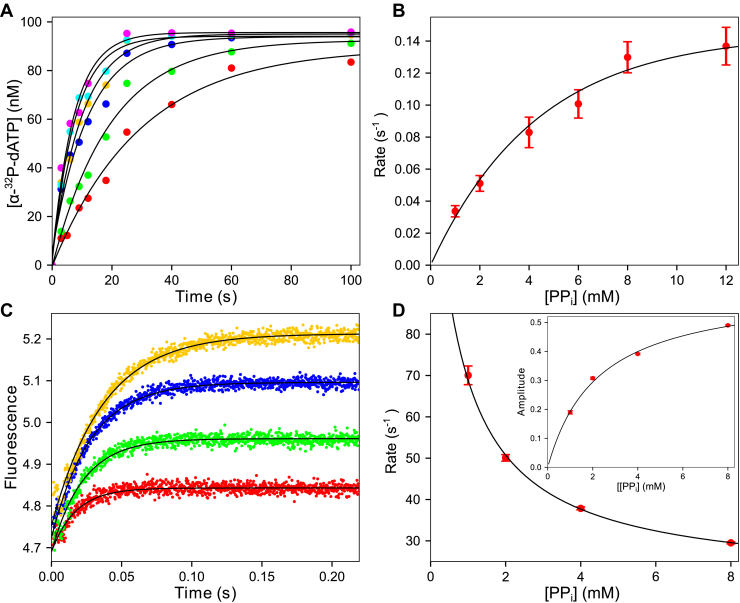
**Kinetics of pyrophosphorolysis.***A*, hand quench pyrophosphorolysis with 3’-[α- ^32^P]-labeled DNA. A solution of T7 DNA polymerase E514Cou (500 nM), thioredoxin (10 μM), BSA (0.1 mg/ml), 3’-[α-^32^P]-28/45 DNA (100 nM), and PP_i_ (1–12 mM) was mixed with unlabeled dATP (20 μM) and Mg^2+^ (12.5 mM) to start the reaction. Data at each PP_i_ concentration are shown fit to a single exponential function (*black lines*). *B*, rate *versus* PP_i_ concentration for pyrophosphorolysis experiment. Rates are from the data in (*A*). Data are shown fit to a hyperbola (*black line*), estimating an apparent *K*_*d,PPi*_: 5.6 ± 1.2 mM and *k*_*PPi*_ 0.20 ± 0.02 s^−1^. *C*, stopped flow pyrophosphorolysis T7 DNA polymerase E514Cou (500 nM), thioredoxin (10 μM), 28/45 DNA (600 nM), and PP_i_ (1–8 mM) were mixed with Mg^2+^ (12.5 mM) to start the reaction. Data at each PP_i_ concentration were fit to a single exponential function (*black lines*). *D*, rate and amplitude *versus* PP_i_ concentration for stopped flow pyrophosphorolysis. Rates and amplitudes are from data in (*C*). Data are shown fit to a hyperbola (*black line*) estimating an apparent *K*_*d,PPi*_: 950 ± 390 μM. *Inset:* Amplitude *versus* PP_i_ concentration. Shown fit to a hyperbola (*black line*), estimating an apparent *K*_*d*_ of 2.2 ± 0.3 mM. Increasing amplitude with increasing PP_i_ concentration is indicative of a "conformational selection" mechanism.

A preformed E-[α-^32^P]-28/45 DNA complex with varying concentrations of PP_i_ (1–12 mM) was mixed with Mg^2+^ and unlabeled dATP (20 μM) to start the reaction. Reactions were quenched at various times by adding 0.3 M EDTA, and reaction products were analyzed by TLC on PEI-cellulose plates to resolve the formation of the product [α-^32^P]-dATP (Fig. 4*A*).

Unlabeled dATP served as a trap to prevent the rebinding of [α-^32^P]-dATP and to fill in any primer where the radiolabeled dAMP has been removed. This prevents multiple rounds of pyrophosphorolysis, which could produce dNTPs that would then serve as substrates for correct incorporation on top of the radiolabeled 3’ dAMP of the primer strand. The low concentration of dATP used in the experiment was chosen to preclude incorporation of a dATP:dA mismatch on top of the [α-^32^P]-labeled primer strand (11) but is sufficient to rapidly replace the [α-^32^P]-dAMP after its removal from the primer. These details allow the experiment to be interpreted without complications. In addition, although Mg^2+^-PP_i_ precipitates at concentrations above ∼5 mM, the precipitation occurs on the timescale of approximately 30 s, whereas the pyrophosphorolysis reactions at concentrations of Mg^2+^-PP_i_ ≥ 5 mM occurs on a shorter timescale. Therefore, measurements can be made before precipitation by mixing PP_i_ (without Mg^2+^) and the E-[α-^32^P]-28/45 complex with Mg^2+^ to start the reaction. A control stopped flow experiment showed PP_i_ does not bind to the enzyme in the absence of Mg^2+^ (not shown).

The time dependence of forming [α-^32^P]-dATP (Fig. 4*A*) at each PP_i_ concentration was adequately fit using a single exponential function (Equation 3). The PP_i_ concentration dependence of the observed rate (Fig. 4*B*) was fit to a hyperbola to give an apparent *K*_*d,app*_ = 5.6 ± 1.2 mM for PP_i_ and a maximum rate of pyrophosphorolysis, *k*_*PPi*_ = 0.2 ± 0.02 s^−1^. The weak apparent binding affinity and slow rate of pyrophosphorolysis indicate that the reverse reaction is highly unfavorable relative to the forward dATP incorporation reaction. However, these parameters underestimate the efficiency of pyrophosphorolysis owing to additional aspects of the reaction, such as translocation, which can be resolved by global data fitting including additional experiments.

#### Conformational changes during pyrophosphorolysis

We reasoned that, since there is a fluorescence signal for the forward reaction, there should also be a fluorescence signal associated with PP_i_ binding upon forming the *FD*_*n*_*N* and *FD*_*n+1*_*.PP*_*i*_ states that can be followed by stopped-flow methods (Equation 1). We performed this experiment (Fig. 4*C*) by mixing a solution containing the *E.DNA* complex and varying concentrations of PP_i_ (1–8 mM) with Mg^2+^ to start the reaction. Data collection was limited to 250 ms to restrict the observed portion of the reaction to PP_i_ binding. On a longer timescale, a subsequent forward reaction after dATP is produced leads to a more complex signal.

The stopped flow traces at all PP_i_ concentrations fit a single exponential function (Equation 3). Fitting the rate and amplitude *versus* PP_i_ concentration using a hyperbola is shown in Figure 4*D*. Of interest, the observed rate of the fluorescence transient decreased with increasing PP_i_ concentrations with an *apparent K*_*d*_ = 950 ± 390 μM. However, the amplitude increased. This type of behavior is the kinetic signature for a “conformational selection” mechanism (25, 26), where a rate-limiting isomerization of the enzyme occurs before binding of the substrate (PP_i_ in this case). In particular, it is important that attempts to fit the data by simulation with all experiments up to this point using the model in Equation 1 were unsuccessful because they failed to provide a model containing two enzyme states prior to PP_i_ binding (*i.e.*, translocation).

**Figure 5:**
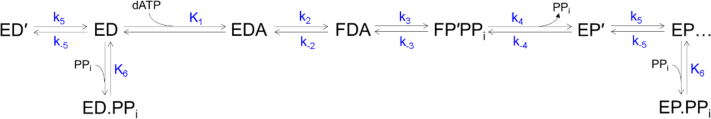
**Full kinetic model for nucleotide incorporation by T7 DNA polymerase.** This pathway is expanded from Equation 1 to Include translocation steps (*k_5_*, *k_-5_*) and competitive PP_i_ binding to the post-translocated state (*K_6_*).

A reasonable fit to the data was obtained by adding a translocation step (ED′→ ED or EP′→ EP) after PP_i_ release but before binding of the next dNTP (Fig. 5).

As the translocation step is the only known step in the polymerization cycle that is not included in the model in Equation 1, we hypothesized that translocation could modulate the binding of dNTP or PP_i_ as first described in prior work on the T7 DNA polymerase (11, 14). The concept of binding of PP_i_ or dNTP to a pre-existing equilibrium between pre- and posttranslocation states, respectively, was later given the name of a “Brownian ratchet” (27). In this model, the release of the PP_i_ product is separated from dATP binding by a reversible translocation step. After translocation, ATP binding traps the DNA at the active site. This contrasts with the “power-stroke” model where PP_i_ and dNTP bind to the same enzyme state, and subsequent to dNTP binding translocation and PP_i_ release occur simultaneously (28).

Our updated model including translocation is shown in Figure 5, where *k*_*5*_ is the rate constant governing translocation from the pretranslocated states (*EP*′, *ED*′ that are able to bind PP_i_) to the posttranslocated states (*ED*, *EP* that are able to bind the next incoming nucleotide). Because the equilibrium favors the translocated state, dATP binds to the predominant state in the population and the subsequent conformational change follows an *induced-fit model*. In contrast, PP_i_ binds only to the minor (untranslocated) state following a pattern of *conformational selection*.

With this more complete model, extrapolation of the observed rate *versus* concentration plot to zero [PP_i_] (Fig. 4*D*) provides an estimate for the sum *k*_−*5*_ + *k*_*5*_. Although there are large errors on this y-intercept because of the decreasing amplitude with decreasing concentrations of PP_i_, the data are sufficient to set a lower limit of ∼100 s^−1^ for the sum of these two rate constants (*k*_−*5*_ + *k*_*5*_). The rate constant for reverse translocation (*k*_−*5*_) is better defined because it is obtained using data at higher PP_i_ concentrations, extrapolated to give *k*_−*5*_ ∼10 s^−1^.

The experiments in the next section were designed to more rigorously distinguish the model in Equation 1 from the model in Figure 5.

### Inhibition of dATP incorporation by PP_i_

The model in Figure 5 has some important and testable implications that distinguish it from the power stroke model where the nucleotide and PP_i_ bind to the same enzyme state. The following experiments were designed using KinTek Explorer with the two different models to determine the best reaction conditions to differentiate the two kinetic schemes. The first experiment involves performing the forward nucleotide incorporation reaction in the presence of varying concentrations of PP_i_.

#### Stopped flow PP_i_ inhibition on dATP incorporation

**Figure 6:**
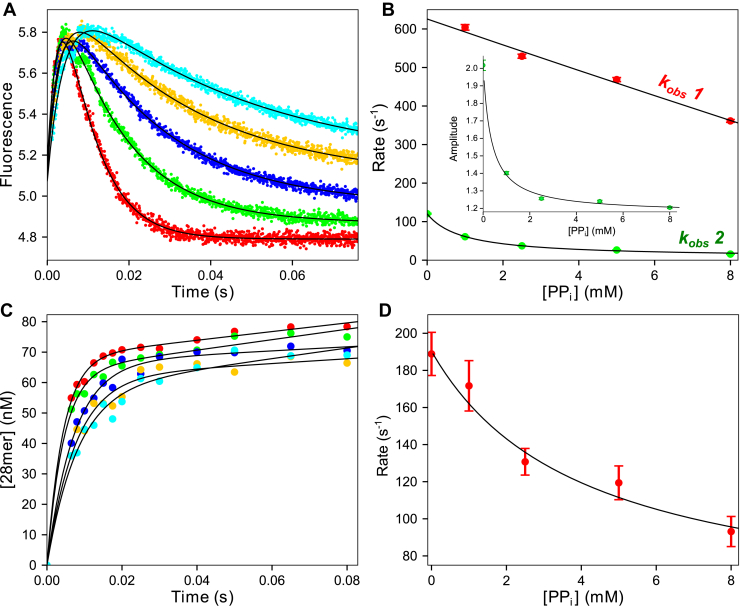
**Kinetics of PP**_**i**_**inhibition on dATP incorporation.***A*, fluorescence measurement during PP_i_ inhibition on dATP incorporation. T7 DNA polymerase E514Cou (500 nM), thioredoxin (10 μM), 27/45 DNA (600 nM), and PP_i_ (0–8 mM) were mixed with dATP (50 μM) and Mg^2+^ (12.5 mM) to start the reaction. Data at each concentration were fit to a double exponential function (*black lines*). *B*, rate *versus* PP_i_ concentration for inhibition of dATP incorporation. Rates are from data in (*A*). Data are shown for the fast phase (*red*, *k*_obs_ 1) and slow phase (*green*, *k*_obs_ 2), fit to a line and a hyperbola, respectively (*black lines*). The slope of the linear fit of the slow phase derived from the fitting is −0.028 ± 0.003 mM^−1^ s^−1^, which suggests weak competitive inhibition by PP_i_ for binding to the enzyme. The hyperbolic fit to the slow phase gives an apparent *K*_*d,PPi*_: 775 ± 75 μM. *Inset:* Amplitude *versus* PP_i_ concentration for the slow phase, estimating an apparent *K*_*d*_ of 350 ± 80 μM. *C*, quench flow measurement of PP_i_ inhibition of dATP incorporation. T7 DNA polymerase E514Cou (120 nM), thioredoxin (2.4 μM), FAM-27/45 DNA (200 nM), BSA (0.1 mg/ml), and PP_i_ (0–12 mM) from one syringe were mixed with dATP (20 μM) and Mg^2+^ (12.5 mM) from the other syringe to start the reaction. Data at each PP_i_ concentration are shown fit to a burst equation (*black lines*). *D*, rate *versus* PP_i_ for inhibition on dATP incorporation. Rates are from data in (*C*). Data for the exponential phase of the burst at each concentration are shown fit to a hyperbola (*black line*) and gives an apparent *K*_*i,PPi*_: 5.9 ± 3.1 mM.

With the model in Figure 5, inhibition by rebinding PP_i_ directly to the EP’ state formed after chemistry should appear to be much tighter than the affinity derived from the experiment in Figure 4*A* where PP_i_ binding must overcome the unfavorable translocation equilibrium. To test this hypothesis, we monitored the stopped-flow fluorescence signal observed at a fixed dATP concentration and varying concentrations of PP_i_. In this experiment a preformed *E.DNA* complex with varying concentrations of PP_i_ (0–8 mM) was mixed with Mg^2+^ and a fixed concentration of dATP (50 μM) in the stopped flow to start the reaction (Fig. 6*A*). Traces at each PP_i_ concentration were fit to a double exponential function, and the observed rates for the fast and slow phases were plotted *versus* PP_i_ concentration (Fig. 6*B*). It is surprising that the rate of the fast phase, corresponding to dATP binding steps, showed only a slight inhibition with increasing PP_i_ concentrations (step 6 in Fig. 5). This inhibition of the fast phase fits a linear function with a slope of −0.027 ± 0.003 mM^−1^ s^−1^. The concentration dependence on the rate of the isomerization accompanying PP_i_ release following chemistry showed an apparent *K*_*d*_ = 775 ± 75 μM, which is comparable with that obtained with the stopped flow pyrophosphorolysis experiment in Figure 4*C*, consistent with the translocation model shown in Figure 5.

Fitting this experiment globally in KinTek Explorer with all experiments presented up to this point also required an additional rapid equilibrium PP_i_ inhibition step (*K*_*6*_), competitive with dNTP, that binds to the posttranslocated state (*ED, EP*) with a *K*_*i*_ of approximately 6 mM (Figure 5). This accounts for the small linear inhibition of the rate of the fast phase, in addition to the translocation step proposed earlier. Addition of this competitive inhibition step did not significantly change the rate constants for nucleotide binding (*K*_*1*_*, k*_*2*_*, k*_−*2*_), chemistry (*k*_*3*_), or the reverse of translocation (*k*_−*5*_); however, the reverse of chemistry (*k*_−*3*_) was increased ∼3 fold to 136 s^−1^, requiring also a small increase in *k*_*4*_ to 220 s^−1^ and an increase in *k*_*5*_ to ∼200 s^−1^ to achieve an optimal global fit of the data, which is described below.

#### Quench flow measurement of PP_i_ inhibition of dATP incorporation

A parallel experiment was performed in the quench flow by mixing a preformed *E.DNA* complex and varying concentrations of PP_i_ (0–8 mM) with Mg^2+^ and dATP (20 μM) to start the reaction (Fig. 6*C*). The data at each PP_i_ concentration were fit to a burst equation (Equation 6), and the observed rate of the exponential phase *versus* PP_i_ concentration is shown fit to a hyperbola in Figure 6*D*. Minimal inhibition is observed as the majority of the enzyme species is in the state ready to bind dATP (*ED*) with only weak competitive PP_i_ inhibition, not the form (*ED*′) that tightly binds PP_i_. PP_i_ does not significantly inhibit steps until after the product DNA has been formed and is committed to going forward to form product in steps that are not observed in this experiment.

This experiment was performed in the original series of papers on this enzyme (14) and is consistent with the work presented here where PP_i_ acts as a weak competitive inhibitor for nucleotide binding with an observed effect on the rate of the burst. This experiment does not provide information to justify one translocation model over the other. Rather, it is included for consistency with previously published work and shows the additional information obtained from using a labeled protein to dissecting the reaction to give the complete story.

### Kinetics of processive polymerization

The model proposed in Figure 5 has important implications for processive polymerization. The first prediction, mentioned earlier, is that incorporation of nucleotides after the first incorporation may be slower than the first incorporation owing to rate limiting PP_i_ release and translocation in the reaction pathway. The second prediction is that PP_i_ will inhibit the second nucleotide incorporation in a series to a much greater extent than the first incorporation. After the first incorporation, the ED complex will pass directly through the state that is able to bind PP_i_ (*ED*′_*28*_ in Equation 8) with relatively high affinity. These two predictions are tested with the following experiment.

(8) $$\begin{array}{l} ED_{27}\overset{+dATP}{\to }F{D_{28}}^{\prime }PP_{i}\underset{PP_{i}}{⇄}ED_{28}^{\prime }\overset{trans.}{⇄}ED_{28}\\ ED_{28}\overset{+dTTP}{\to }F{D_{29}}^{\prime }PP_{i}\underset{PP_{i}}{⇄}ED_{29}^{\prime }\overset{trans.}{⇄}ED_{29} \end{array}$$

#### Processive polymerization and inhibition by PP_i_

**Figure 7:**
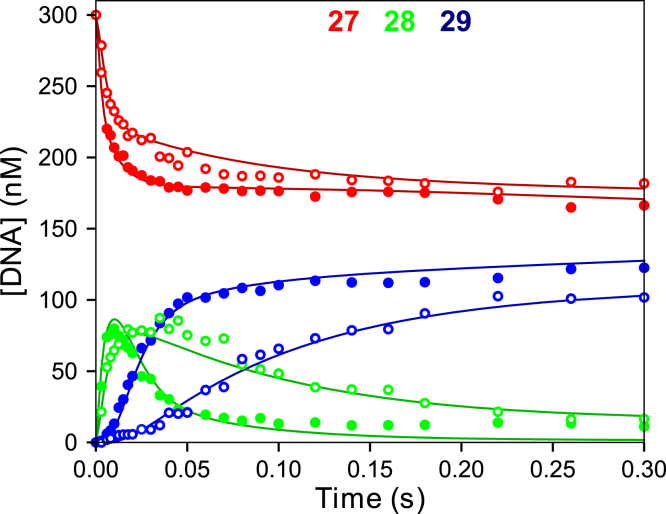
**PP**_**i**_**inhibition on processive polymerization.** T7 DNA polymerase E514Cou (120 nM), thioredoxin (4 μM), FAM-27/45 DNA (300 nM), BSA (0.1 mg/ml), and PP_i_ (0 or 8 mM) were mixed with dATP (100 μM), dTTP (100 μM), and Mg^2+^ (12.5 mM) to start the reaction. DNA lengths for each color are given at the top of the panel. The closed data points were collected without added PP_i_, and the open data points had 8 mM PP_i_ in the reaction. The rates for the first and second incorporations for the simple model ED_27_→ED_28_→ED_29_ for the reaction without PP_i_ were 182 ± 10 s^−1^ and 41 ± 1 s^−1^, respectively. The rates derived from the fit by simulation for the two incorporations for the reaction with 8 mM PP_i_ are 104 ± 8 s^−1^ and 9.3 ± 0.6 s^−1^, respectively. Lines through the data were computed based on global data fitting shown in Figure 8.

To test these two hypotheses, we performed a rapid quench experiment where an *E.DNA* complex, in the presence or absence of 8 mM PP_i_, was mixed with dATP (100 μM), dTTP (100 μM), and Mg^2+^ to start the reaction (Fig. 7). Fitting the data for the experiment without PP_i_ (closed circles) gives net rates of 182 ± 10 and 41 ± 1 s^−1^ for the first and second incorporations, respectively, based on a simple two-step reaction: ED_27_ → ED_28_ → ED_29_. The second incorporation is much slower than the first incorporation, owing to the rate-limiting PP_i_ release and translocation steps that occur after fast chemistry for the first incorporation (Equation 8). The experiment performed in the presence of 8 mM PP_i_ (open circles) shows some inhibition of the rate of the first incorporation, with a rate of 104 ± 8 s^−1^, whereas the rate of the second incorporation, 9.3 ± 0.6 s^−1^, was significantly reduced. For the simple model in Equation 1, even at 8 mM, one would expect only slight inhibition on the second incorporation (less than 2-fold). The large inhibition of the second incorporation reaction provides further support for the model in Figure 5 and Equation 8.

### Global fitting of data and free-energy profile

**Figure 8:**
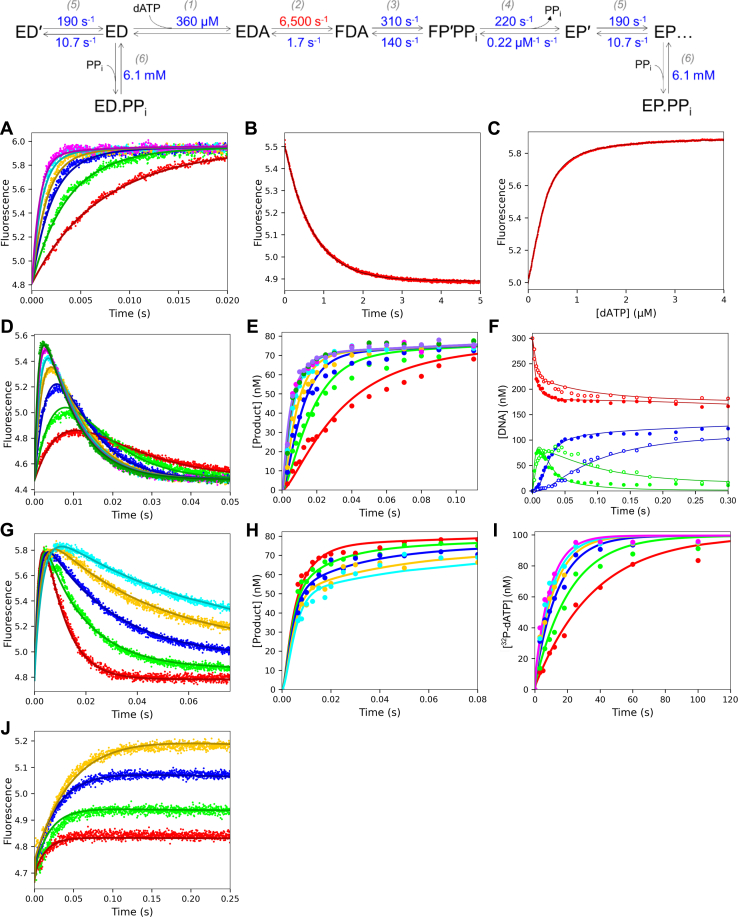
**Global fit of all experimental data.** The scheme at the top contains the rate constants derived from the global fit of the data shown in this figure. Error estimates for each rate constant are given in Table 1. Rate constants in *red* were locked during the fitting, and the numbers in parenthesis above the steps are the step numbers. The experiments in Figure 2, Figure 3, Figure 4, 6 and 7 are shown with solid colored lines through the data showing the global fit to the rate constants in the scheme at the top of the figure. *A*, stopped flow dATP binding rate. *B*, stopped flow dATP dissociation rate. *C*, equilibrium dATP binding titration. *D*, stopped flow fluorescence during dATP incorporation. *E*, quench flow measurement of dATP incorporation. *F*, quench flow measurement of PP_i_ inhibition of processive polymerization. *G*, stopped flow measurement during PP_i_ inhibition of dATP incorporation. H, quench flow measurement of PP_i_ inhibition of dATP incorporation. *I*, kinetics of pyrophosphorolysis. *J*, stopped flow pyrophosphorolysis.

**Figure 9:**
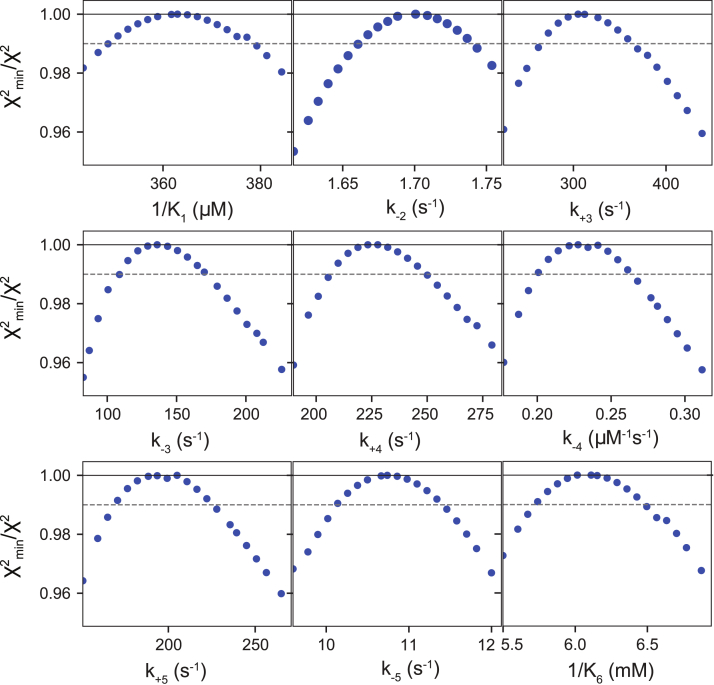
**Confidence contour plots for rate constants in the dATP incorporation pathway.** The confidence contours from the 1D FitSpace calculation in KinTek Explorer are shown as *blue data points* for each rate constant. The *gray dashed line* shows the 0.99 χ^2^ threshold to establish 95% confidence intervals for each rate constant, calculated with KinTek Explorer and summarized in Table 1.

All experiments were globally fit in KinTek Explorer (29, 30) using the unified model shown in Figure 5. The rate constants for each step in the pathway derived from the global fitting are given in the scheme at the top of Figure 8, and the error estimates are listed in Table 1. The global fit for all experiments is shown as the solid-colored lines in Figure 8. The confidence contour FitSpace analysis (24) was used to provide the most rigorous statistical test to determine whether each parameter in the model was well constrained by the experimental data. The value of χ^2^ is plotted *versus* the systematically varied value for each rate constant as shown in Figure 9. Of importance, the error profile for each rate constant is symmetric showing that each is well constrained by the data.

**Table 1:** Rate constants derived by global data fitting

Parameter	Best-fit value	95% Confidence interval
*K*_*1*_*k*_*2*_	18 ± 0.6 μM^−1^ s^−1^	17.4–18.6
1/*K*_*1*_	360 ± 12 μM	351–377
*k*_*2*_	[6500 s^−1^]	≥6000
*k*_−*2*_	1.70 ± 0.04 s^−1^	1.67–1.74
*k*_*3*_	310 ± 40 s^−1^	273–359
*k*_−*3*_	140 ± 30 s^−1^	115–170
*k*_*4*_	220 ± 18 s^−1^	210–246
*k*_−*4*_	0.22 ± 0.03 μM^−1^ s^−1^	0.201–0.261
*k*_*5*_	190 ± 25 s^−1^	171–222
*k*_−*5*_	10.7 ± 0.6 s^−1^	10.1–11.3
*K*_*6*_	6.1 ± 0.3 mM	5.74–6.43

Lower and upper limits are reported from a 0.99 χ^2^ threshold to give a 95% confidence interval. The value of *k*_2_ (in brackets) was locked during fitting, which determines the value for *K*_1_. For each of the two equilibrium constants (*K_1_* and *K_6_*), the binding rate constant was locked and fitting to define the dissociation rate constant was used to estimate the equilibrium constant. For example, *k*_*1*_ was locked at 100 μM^−1^ s^−1^ and *K*_*1*_*= k*_*1*_*/k*_−*1*_ was calculated from estimates for *k*_−*1*_ derived in fitting. Similarly, *K*_*6*_ = *k*_*6*_*/k*_−*6*_ with *k*_*+6*_ locked in fitting.

The new standard for finding a minimal model is based on defining a pathway that is necessary and sufficient to account for all available data. Achieving a good fit establishes that the minimal model is sufficient to account for the data. Showing that all kinetic parameters are well constrained by the data establishes that the model is not overly complex (30).

**Figure 10:**
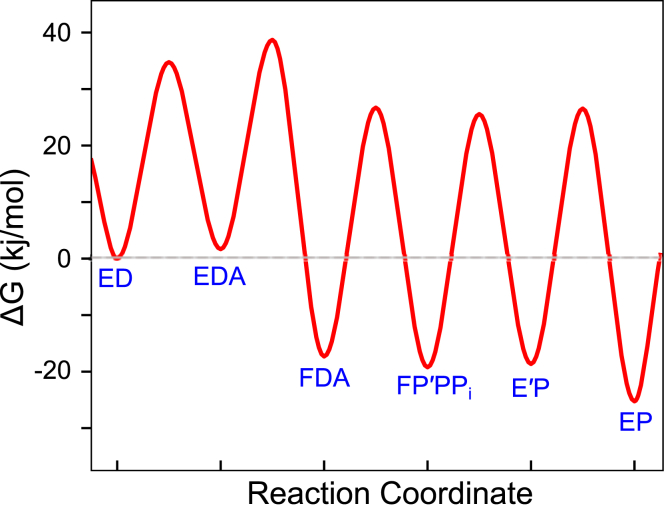
**Free-energy profile for dATP incorporation at 4 °C.** The free-energy profile was generated using the rate constants and model in the scheme at the top of Figure 8, at 277 K, and physiological concentrations of substrate and product (175 μM dATP, 1.3 mM PP_i_). A transmission coefficient of 0.01 was used to better display the relationships.

Table 1 shows the best-fit values for each rate constant along with upper and lower limits derived from the confidence contour analysis with a χ^2^ threshold of 0.99. From the mechanism and rate constants in Figure 8 and physiological dATP and PP_i_ concentrations, a free-energy profile was created in KinTek Explorer (Fig. 10). Most importantly, the specificity-determining step is the highest barrier relative to the starting material, which is the conformational change step. There is no single rate-determining step because *k*_*3*_, *k*_*4*_, and *k*_*5*_ are comparable.

The complete solution to derive steady-state parameters from intrinsic rate constants for the model shown in Figure 5 is:

$$\begin{array}{l} k_{cat}=\frac{k_{2}k_{3}k_{4}k_{5}}{k_{-3}k_{5}\left(k_{2}+k_{-2}\right)+k_{4}k_{5}\left(k_{2}+k_{-2}+k_{3}\right)+k_{2}k_{3}\left(k_{4}+k_{5}\right)}\\ k_{cat}/K_{m}=\frac{k_{1}k_{2}k_{3}k_{4}k_{5}}{\left(k_{5}+k_{-5}\right)\left\{k_{-1}k_{-2}\left(k_{-3}+k_{4}\right)+k_{3}k_{4}\left(k_{-1}+k_{2}\right)\right\}} \end{array}$$

Using these equations and the rate constants in Table 1, we calculate *k*_*cat*_ = 75 s^−1^ and *k*_*cat*_/*K*_*m*_ = 16.5 μM^−1^ s^−1^ for processive synthesis, partially rate limited by PP_i_ release and translocation. Based on a rapid equilibrium nucleotide binding model the specificity constant can be approximated as:

$$K_{cat}/K_{m}\approx K_{1}k_{2}k_{5}/\left(k_{5}+k_{-5}\right)$$

The conformational change after weak ground-state nucleotide binding is the primary determinant of specificity *via* this induced-fit mechanism but is attenuated by the fraction of ED complex that is in the translocated state (95%).

## Discussion

The work presented here provides the first fully complete kinetic analysis of correct nucleotide incorporation by a high-fidelity DNA polymerase. Global data fitting was required to achieve a complete fit of the full data set to support a single unifying model. It is difficult to reconcile the results of conventional equation-based data fitting with the intrinsic rate constants given in the scheme in Figure 8 because the simplifying assumptions required to derive equations are inaccurate for this complex model. For example, the nucleotide concentration dependence of the rate of incorporation (Fig. 3, *A*–*B*) gave a maximum polymerization rate, *k*_*pol*_ = 210 s^−1^, and an apparent *K*_*d,app*_ value of 15.5 μM providing an estimate of *k*_*pol*_*/K*_*d,app*_ = 13.5 μM^−1^ s^−1^. The specificity constant defined by global fitting was somewhat higher, *k*_*cat*_*/K*_*m*_ = 17 μM^−1^ s^−1^, whereas the rate constant for the chemical reaction was significantly higher than the observed rate of the burst, *k*_*3*_ = 312 s^−1^. The solution of a simple burst equation implies that the observed decay rate should be equal to the sum of all rate constants contributing to the endpoint. However, based on global data fitting we now know that the pre-steady-state burst of incorporation will not follow a single exponential; rather, it is a sum of two or three exponentials with comparable rate constants that blend together to give an observed decay rate that can be adequately fit using a single exponential function with a decay rate that is slower than the individual rate constants. Certainly, quench flow data with few data points are not sufficient to resolve multiple exponentials. Similarly, measurements of the kinetics of pyrophosphorolysis yielded results that are attenuated by translocation, the conformational change, and the reversible chemical reaction. In globally fitting data from all experiments, the underlying model and intrinsic rate constants are resolved by taking into account the way in which different aspects of the pathway affect a given measurement designed to measure a single step.

The apparent *K*_*d,PPi*_ from the rate *versus* concentration plot in Figure 4*D* is 950 ± 390 μM, which is defined in terms of rate constants as *K*_*d,PPi*_ = *k*_*4*_(*k*_*5*_ + *k*_−*5*_)/*k*_−*4*_*k*_*5*_, which approximates the true *K*_*d*_ for PP_i_ binding to the pretranslocated state. Given estimates for *k*_*4*_ (125 s^−1^ from Fig. 3), *k*_−*4*_, or the second-order rate constant for PP_i_ binding to the pretranslocated state, can be estimated around 0.2 μM^−1^ s^−1^. When compared with the value for *k*_−*4*_ in fitting to Equation 1 (∼0.02 μM^−1^ s^−1^), with unchanged *k*_*4*_, this model predicts PP_i_ binding ∼10-fold tighter to the enzyme than previously measured from the hand quench experiment owing to pre-equilibration of the enzyme into the posttranslocated state (EP) that is unable to bind PP_i_ that must be overcome before the reverse reaction can proceed.

We show that, for our fluorescent DNA polymerase (21), initial weak ground-state nucleotide binding (1/*K*_*1*_ ≥ 360 μM) induces a conformational change (*k*_*2*_, ≥ 6000 s^−1^) that is much faster than the rate of chemistry (*k*_*3*_ = 310 s^−1^). Although neither *K*_*1*_ nor *k*_*2*_ is well defined, the apparent second-order rate constant for nucleotide binding is well defined as *K*_*1*_*k*_*2*_ = 18 ± 0.6 μM^−1^ s^−1^ (Fig. 2*C*). Of importance, the rate constant for reverse of the conformational change step allowing nucleotide release (*k*_−*2*_ = 1.7 s^−1^) is much slower than chemistry (Fig. 2*D*). Thus, kinetic partitioning of the *FDN* state to either release nucleotide or form product for the correct nucleotide greatly favors the forward reaction. Specificity is a function of all steps leading up to the first largely irreversible step, which can be identified as the highest overall barrier in the free-energy profile (Fig. 10). Accordingly, the specificity constant (*k*_*cat*_/*K*_*m*_) is determined solely by the net second-order rate constant for binding defined by product of the equilibrium constant for nucleotide binding to the open state times the rate of the conformational change (1, 17).

Prior studies on an MDCC-labeled 8-Cys lite T7 DNA polymerase variant (31) provided an estimate of the rate of the conformational change of 700 s^−1^ at 20 °C, a similar nucleotide dissociation rate of 2 s^−1^, a similar rate of chemistry around 300 s^−1^, but a higher-affinity ground-state binding at 28 μM (1). The extensive mutagenesis required for site-specific cysteine labeling decreased the fidelity of this enzyme variant by greatly increasing the ground-state binding affinity of mismatches. In comparison, a single unnatural amino acid variant of T7 DNA polymerase maintains the fidelity of the wildtype enzyme (21). Although the ground-state binding affinity is approximately 10-fold weaker for our variant, the rate of the conformational change is at least 10 times faster, in spite of the lower temperature in this study. One study using microfluidics provided an estimate of the substrate-induced conformational change of 4700 s^−1^ but the effect of the Cy3 label in this study was not examined (32).

In studies on HIVRT, another family A polymerase, a fluorescently labeled variant (17) showed similar rate constants with weak ground-state binding (200 μM), a fast conformational change (2000 s^−1^), but a slower nucleotide dissociation rate (0.06 s^−1^) and chemistry (15 s^−1^). In both HIVRT and T7 DNA polymerase, the conformational change is the specificity-determining step for correct nucleotide incorporation, as it is the highest barrier relative to the starting material (Fig. 10).

Studies on polymerase β (33) also revealed a nucleotide-induced conformational change at 1400 s^−1^ but a much slower rate of chemistry (6.4 s^−1^) and faster nucleotide dissociation rate (12 s^−1^). For this low-fidelity repair enzyme, chemistry is the predominant specificity-determining step as it represents the highest overall barrier, but *k*_*cat*_/*K*_*m*_ is also affected by the two-step rapid-equilibrium nucleotide binding. Because *k*_−*2*_ > *k*_*3*_, the specificity constant is approximately defined by *k*_*cat*_*/K*_*m*_ ≈ *K*_*1*_*K*_*2*_*k*_*3*_.

In this report a single unnatural coumarin amino acid afforded an optimal signal to monitor conformational dynamics during DNA polymerization. The rise and fall of the fluorescence transient during a single round of nucleotide incorporation (Fig. 3*C*) revealed the closing of the enzyme after binding nucleotide followed by chemistry (measured by rapid quench in Fig. 3*A*) then reopening to release the PP_i_ product. Globally fitting fluorescence transients with rapid-chemical quench flow data allows resolution of each step in the pathway by correlating conformational changes with measurable chemical reactions. Under our experimental conditions at 4 °C, we showed that enzyme reopening (and presumably PP_i_ release) occurred at a rate that is slower than the chemistry and therefore limits the rate of processive DNA replication. This conclusion was confirmed by measurements of the effect of added pyrophosphate on processive synthesis (Fig. 7).

Similar studies on DNA polymerase β measuring the fluorescence transient gave a rate of PP_i_ release that was coincident with chemistry, signifying that the chemistry was the rate-limiting step in the pathway and PP_i_ release is fast (33). Similarly, when HIVRT is copying a DNA template, the kinetics of the chemical reaction, enzyme opening (measured by fluorescence), and PP_i_ release are all coincident, suggesting that the rate-limiting chemistry is followed by rapid enzyme opening and PP_i_ release (17, 34). However, for an RNA template, chemistry is fast (250 s^−1^) and PP_i_ release is slow (58 s^−1^), which limits the rate of processive synthesis (35), as seen for T7 DNA polymerase at 4 °C.

To derive the most complete model we also performed experiments on the reverse (pyrophosphorolysis) reaction using both chemical quench methods as well as stopped flow methods to monitor conformational change using our labeled T7 DNA polymerase variant. Interestingly, when fluorescence changes were measured during pyrophosphorolysis, the traces showed an increasing amplitude but decreasing rate with increasing PP_i_ concentrations (Fig. 4*C*), indicative of a “conformational selection” mechanism. We showed that these results could be explained by an unfavorable reverse translocation step required before PP_i_ could bind to the less populated ED′ state. These experiments help to define the kinetics of the translocation step from ED′ to ED with an equilibrium constant of *K*_*5*_ = 18 in the forward direction.

$$ED^{\prime }\overset{188\;s^{-1}}{\underset{11\;s^{-1}}{⇄}}ED$$

In this model, PP_i_ release occurs with enzyme opening to form ED′ and is followed by translocation. Similar ensemble measurements could potentially be performed on other labeled DNA polymerases to extract translocation rates. This sequential reaction is counter to the predictions of the power stroke model. Initial structural studies suggested a power stroke model for T7 RNA polymerase (36), but direct measurement of the kinetics of translocation by ensemble methods has proven difficult.

Of importance, our new data define the rate constants for translocation during DNA polymerization. Prior measurements of translocation have relied on single molecule methods, using optical tweezer experiments or experiments using an α-hemolysis nanopore (22, 23) at various forces or potential differences, respectively. These data suggest a rapid equilibrium constant for translocation with rate constants on the order of 1000 s^−1^ and an equilibrium constant near unity. This represents an example of a Brownian ratchet in which translocation is driven by selective binding to the posttranslocation state, which is in rapid equilibrium with the pretranslocation state. In contrast, our data present a different view in which translocation is favored thermodynamically (*K*_*6*_ = 18, ΔG = −6.7 kJ/mol at 4 °C) but is also rate limiting during processive polymerization. Thus, measured rates of translocation during polymerization do not support either the Brownian ratchet or the power stroke model, each of which invokes nucleotide binding to drive translocation. Although nucleotide binding to the posttranslocation state could drive the translocation reaction to completion (14), we now know that the translocation is not in rapid equilibrium. Rather, the translocation proceeds in the absence of nucleotide to 95% completion to facilitate rapid nucleotide binding to the posttranslocation state. In our experiments performed at 4 °C, translocation was rate limiting, allowing direct measurement of the rate. Experiments remain to be performed to see how the rate constants change at higher temperatures, where the rate of translocation is expected to increase.

All experiments were performed at 4 °C, which is a lower temperature than optimal, but certainly within the physiological range for *Escherichia coli*. Work on other polymerases has shown that fidelity does not seem to change much as a function of temperature (37). However, the rates of the conformational change for HIVRT showed a significant temperature dependence, which afforded accurate estimates of the rate of the conformational change by extrapolation to 37 °C (17, 38). Differences in temperature dependence are likely to reflect the physiologically important range of temperatures for a particular enzyme. While HIVRT evolved to infect human cells at 37 °C, presumably T7 bacteriophage must have evolved to infect *E. coli* over a wide range of temperatures. The rate of nucleotide incorporation is too fast to measure accurately at 20 °C, but the temperature dependence of the experiments presented here would allow fast rates to be better estimated by extrapolation to higher temperatures. Such measurements would also provide valuable enthalpy and activation energies for each step in the pathway.

The data reported here provide the first complete pathway and free-energy profile for a correct nucleotide incorporation for a high-fidelity DNA polymerase. This work extends our understanding of the induced-fit mechanism described for the HIVRT DNA polymerase, an enzyme with moderate fidelity. For the full understanding of DNA polymerase fidelity, the misincorporation and exonuclease proofreading reactions must also be quantified. Similar studies on the misincorporation reaction with our labeled T7 DNA polymerase are complex and will be the subject of a future paper.

## Experimental procedures

### Enzyme labeling and purification

We site specifically labeled and purified T7 DNA polymerase with a fluorescent unnatural amino acid, (7-hydroxy-4-coumarin-yl) ethylglycine as described (21). In this report we showed that the labeled enzyme retained activity indistinguishable from that of the wildtype enzyme.

### Preparation of oligonucleotides for kinetics assays

Synthetic oligonucleotides, including oligos containing 2’,3’-dideoxy or 5’-[6-FAM] modifications, were custom synthesized by Integrated DNA Technologies with standard desalting and were further purified in house by denaturing PAGE to >99% full length oligo. Purified oligos were stored in 66.2 buffer (6 mM Tris-HCl pH 7.5, 6 mM NaCl, 0.2 mM EDTA) at −20 °C. Concentrations of oligos were determined by absorbance at 260 nm, using extinction coefficients calculated from the sequence of the oligonucleotide. Extinction coefficients and sequences for each oligo used in this study are listed in Table 2. Primer and template DNA strands were mixed in a 1:1.05 ratio in annealing buffer (10 mM Tris-HCl pH 7.5, 50 mM NaCl, 1 mM EDTA), heated to 95 °C, and cooled slowly to room temperature over approximately 2 h. Double stranded primer/template DNA substrates and their short names used in this paper are shown in Table 3. A control pre-steady-state burst experiment was performed using ^32^P-labeled DNA and FAM-DNA to check for artifacts due to the fluorescent label (not shown). No difference was detected, so the FAM-DNA substrate was used for convenience.

**Table 2:** Oligonucleotides used with extinction coefficients

Oligo name	Sequence (5’→3’)	Extinction coefficient, 260 nm (M^−1^ cm^−1^)
27	CCGTCGCAGCCGTCCAACCAACTCAAC	245,700
27_dd_	CCGTCGCAGCCGTCCAACCAACTCAAC_dd_	245,700
FAM-27	[6-FAM]-CCGTCGCAGCCGTCCAACCAACTCAAC	266,660
28	CCGTCGCAGCCGTCCAACCAACTCAACA	259,500
[α-^32^P]-28	CCGTCGCAGCCGTCCAACCAACTCAAC∗A	259,500
45-18T	GGACGGCATTGGATCGATGTTGAGTTGGTTGGACGGCTGCGACGG	433,700

∗^32^P.

**Table 3:** dsDNA substrates used in kinetics experiments

Name	Sequence
27/45 (DNA)	5’-CCG TCG CAG CCG TCC AAC CAA CTC AAC-3’ 3’-GGC AGC GTC GGC AGG TTG GTT GAG TTG TAG CTA GGT TAC GGC AGG-5’
27_dd_/45 (DNA_dd_)	5’-CCG TCG CAG CCG TCC AAC CAA CTC AAC_dd_-3’ 3’-GGC AGC GTC GGC AGG TTG GTT GAG TTG TAG CTA GGT TAC GGC AGG-5’
28/45	5’-CCG TCG CAG CCG TCC AAC CAA CTC AAC A-3’ 3’-GGC AGC GTC GGC AGG TTG GTT GAG TTG TAG CTA GGT TAC GGC AGG-5’
[α-^32^P]-28/45	5’-CCG TCG CAG CCG TCC AAC CAA CTC AAC∗ A-3’ 3’-GGC AGC GTC GGC AGG TTG GTT GAG TTG TAG CTA GGT TAC GGC AGG-5’
FAM-27/45 (FAM-DNA)	[6-FAM]-CCG TCG CAG CCG TCC AAC CAA CTC AAC-3’ 3’-GGC AGC GTC GGC AGG TTG GTT GAG TTG TAG CTA GGT TAC GGC AGG-5’

∗^32^P.

### Preparation of 3’ [α-^32^P]-dAMP labeled 28/45 DNA substrate

The following components were mixed together in T7 Reaction Buffer (40 mM Tris-HCl, pH 7.5, 50 mM NaCl, 1 mM EDTA, 1 mM DTT) (14) and incubated at room temperature (25 °C) for 5 min: 50 nM wildtype T7 DNA polymerase, 1 μM thioredoxin, 0.1 mg/ml bovine serum albumin (BSA), 1 μM DNA 27/45, 130 nM [α-^32^P]-dATP (Perkin Elmer), and 12.5 mM Mg^2+^. Unlabeled dATP was then added to 10 μM, and the reaction was incubated at room temperature for another 12 min before quenching with the addition of EDTA to 100 mM final concentration. Protein, unincorporated dATP, and [α-^32^P]-dATP were removed using Oligo Clean and Concentrator Spin Columns (Zymo Research). The final product was checked for purity (>95%) and concentration by TLC on PEI-cellulose plates (Sigma) and phosphorimaging.

### Kinetics experiments

All dNTP solutions and BSA were purchased from New England Biolabs. All chemicals used in the reaction buffer were purchased from Fisher Scientific or Sigma-Aldrich. The exonuclease-deficient D5A E7A variant of T7 gene product 5 was used in all experiments and enzymatic synthesis reactions in this paper to prevent exonuclease degradation of the DNA. Wildtype and E514Cou T7 DNA polymerase as well as thioredoxin were expressed and purified as described (21). All reactions were carried out in T7 Reaction Buffer at 4 °C to better resolve the individual rate constants in the pathway. In some experiments the enzyme–DNA complex was preincubated without Mg^2+^ and Mg^2+^-dNTP was added to start the reaction. In other experiments, both reactant mixtures were preincubated in the presence of Mg^2+^ before mixing with dNTP to start the reaction. In both cases, the final total MgCl_2_ concentration after mixing was 12.5 mM and there was no observed difference in the kinetics between the two experimental protocols. All concentrations given are those after mixing.

### Quench flow experiments

Rapid-quench experiments were performed on an RQF-3 instrument (KinTek) with a circulating water bath set to 4 °C. Unless otherwise noted, all experiments were performed with 0.6 M EDTA in the quench syringe and T7 Reaction Buffer without magnesium in the drive syringes. Formamide Loading Buffer (5% sucrose [w/v], 90% formamide [v/v], 0.1% bromophenol blue [w/v], 0.1% xylene cyanol [w/v], 10 mM EDTA) was added to each sample, followed by denaturation at 98 °C for 2 min and separation on 15% polyacrylamide sequencing gels containing 7 M urea for approximately 3 h at 50 °C. Gels were scanned on a Typhoon FLA 9500 laser scanner (GE Healthcare) with the FAM fluorescence filter, and the resulting images were quantified with Image Quant software (GE Healthcare). Concentrations of reaction components given in the text are final concentrations after mixing unless otherwise noted. Quench flow experiments were repeated on separate occasions at least two to three times to ensure reproducibility.

### Stopped flow measurements

All stopped flow experiments were performed on a KinTek SF-300X instrument (dead time of 1.3 ms) with a circulating water bath set to 4 °C. A 150-W Xenon lamp (Hamamatsu) was used as the light source. Samples were excited at 295 nm, and emission was monitored at 445 nm using a filter with a 45-nm band pass (Semrock). All stopped-flow data shown in the main text are an average of at least eight individual traces, and all stopped flow experiments were reproduced at least three times to ensure reproducibility. The TMX titration module for the SF-300X (KinTek Corp) was used for equilibrium titration experiments. Titrations were performed with 280 μl of a solution of enzyme and DNA_dd_ in the cuvette and 20.5 μl of dATP titrant added from a Hamilton syringe over the course of 5 min. The reaction was mixed by constant stirring from a micro-stir bar in the cuvette and the titration data were corrected for the small dilution before fitting in the KinTek Explorer. The titration experiment was performed on three separate occasions to ensure reproducibility.

### Other experiments

For pyrophosphorolysis experiments, a 100 mM stock solution of Na-PP_i_ (Sigma-Aldrich) was prepared, aliquoted, and stored at −80 °C. PP_i_ was incubated in the absence of Mg^2+^ in the mixture containing T7 DNA polymerase for solubility reasons. Control stopped flow experiments (not shown) indicated that PP_i_ does not bind to the enzyme without Mg^2+^, so mixing Mg^2+^ from the other syringe starts the reaction and allows higher concentrations to be reached for short reaction times before Mg^2+^-PP_i_ precipitates. For the experiment with [α-^32^P]-28/45 DNA in Figure 4*A* and the nucleotide dissociation rate experiment in Figure 2*E*, the formation of [α-^32^P]-dATP was monitored by TLC on PEI-cellulose plates. To remove contaminants, TLC plates were first developed in ultrapure H_2_O. Plates were then dried, the contaminants at the top cut off, and the plates stored at 4 °C. Samples were spotted on 1-cm lanes and developed in 0.3 M KPO_4_ pH 7. Plates were then dried, visualized by phosphorimaging, and quantified in Image Quant.

### Kinetics schemes and figures

Structure figures were prepared with the Pymol Molecular Graphics System, Version 2.0 (Schrodinger, LLC). Molecular structures were prepared with the computer program ChemDraw (Perkin Elmer Informatics). Reactions, rate constants, and equilibrium constants in each model are mostly numbered sequentially from left to right, where *k*_*i*_ is the forward rate constant and *k*_−*i*_ is the reverse rate constant for the i^th^ step. The convention for rate constants from previous DNA polymerase kinetics papers (1, 4, 11, 14, 17, 34, 35) was used in Equation 1, where *K*_*1*_ is the equilibrium constant for ground-state nucleotide binding (rapid equilibrium, *k*_−*1*_ >> *k*_*2*_), *k*_*2*_ is the conformational change, *k*_*3*_ is the rate of chemistry, and *k*_*4*_ is pyrophosphate release. Translocation steps added to the final model in Figures 5 and 8 are numbered as step 5 and competitive PP_i_ inhibition is modeled as step 6 to maintain these conventions, although they appear earlier in the model than ground-state nucleotide binding.

### Conventional data fitting

Initially, data from experiments were fit by nonlinear regression with the analytical fit function in KinTek Explorer to single exponential, double exponential, or burst equations given in the main text. Experiments performed at multiple substrate concentrations were fit to one of these equations and the concentration dependence of the observed rate was then either fit to a hyperbola or linear function. The hyperbolic function defines a maximum rate, *k*_*cat*_, and an apparent *K*_*d*_, whereas the linear function defines the apparent second-order rate constant for substrate binding. The patterns in the data and concentration dependence of the rates were important to guide the development of a model for more refined data fitting based on numerical integration of rate equations derived from the full model using the KinTek Explorer software (30). All kinetic data in Figure 2, Figure 3, Figure 4, 6, 7 are shown fit to equations, defined in the main text, where the black line through the data is the fit to the analytical function.

### Data fitting in KinTek explorer

Global data fitting was performed by fitting all experiments in KinTek Explorer to the reaction schemes in the main text, with experimental details of mixing steps and reactant concentrations input for each experiment. In fitting data by simulation, each experiment is modeled exactly as it was performed, including allowing pre-equilibrium of the *E.D* complex. Rapid-quench experiments were modeled simply as the sum of species containing product (*FP.PP*_*i*_*+EP*′*+EP+P*) including product DNA (*P*) release, not shown in the model. Fluorescence transients were modeled by using fluorescence scaling factors. For example, the experiment shown in Figure 3*C* (7D) was modeled as:

$$\text{a∗}\left(\text{E}+\text{E}{\text{D}}^{\prime }+\text{ED}+\text{EDA}+\text{E}{\text{P}}^{\prime }+\text{EP}+\text{b∗}\left({\text{FDA}+\text{FP.PP}}_{\text{i}}\right)\right)$$

where *a* scales the overall signal relative to enzyme concentration and *b* represents the fractional change in fluorescence in forming the closed state. In the process of fitting data, a value of *b* = 1.28 was derived indicating a 28% increase in fluorescence.

For second-order binding steps, only the equilibrium constant for binding was defined by the data (steps 1 and 6 in the scheme shown in Figure 8), so the binding rate constant was locked at 100 μM^−1^ s^−1^ and the reverse rate constant was allowed to float in the fitting to calculate an equilibrium constant. This second-order rate constant represents diffusion-limited substrate binding and is locked at this value to provide a constraint on the rate constants for that step. The confidence contours were derived using the FitSpace function (24). These confidence contour plots are calculated by systematically varying a single rate constant and holding it fixed at a particular value while refitting the data allowing all other rate constants to float. The goodness of fit was scored by the resulting χ^2^ value. The confidence interval is defined based on a threshold in χ^2^ calculated from the F-distribution based on the number of data points and number of variable parameters to give the 95% confidence limits. For the data given in Figure 8, this threshold was of 0.99 and was used to estimate the upper and lower limits for each rate constant.

The free-energy profile in Figure 9 was created in KinTek Explorer using the rate constants given in Table 1 using simple transition state theory.

$$\begin{array}{l} rate=A\cdot \frac{k_{B}T}{h}\exp\left(-\text{Δ}G^{‡}/RT\right)\\ 0<A\leq 1 \end{array}$$

where *k*_*B*_ is the Boltzman constant, *h* is the Planck’s constant, and *R* is the gas constant. The free-energy profile was created using a transmission coefficient of *A* = 0.01 to better show the relationships. Values for the physiological concentrations of dATP (175 μM) (39) and PP_i_ (1.3 mM) (40) were used for each of the second-order rate constants.

## Data availability

All data are contained within the manuscript.

## Conflict of interest

K. A. J. is president of KinTek Corporation, which provided the SF300x stopped-flow and RQF-3 rapid-quench-flow instruments, and KinTek Explorer software used in this study.
